# Partial trisomy 9p22 to 9p24.2 in combination with partial monosomy 9pter in a Syrian girl

**DOI:** 10.1186/1755-8166-3-18

**Published:** 2010-10-04

**Authors:** Walid Al Achkar, Abdulsamad Wafa, Faten Moassass, Thomas Liehr

**Affiliations:** 1 Molecular Biology and Biotechnology Department, Human Genetics Division, Atomic Energy Commission, Damascus, Syria; 2 Jena University Hospital, Institute of Human Genetics, Jena, Germany

## Abstract

**Background:**

Partial trisomy of the short arm of chromosome 9 is among the most common autosomal structural chromosomal anomalies leading to chromosomal imbalance in human. Clinical characteristics are craniofacial dysmorphism including hypertelorism, prominent nose, deep-set eyes, and down-slanting palpebral fissures. The degree of clinical severity in partial trisomy 9p roughly correlates with the size of the chromosomal imbalance. Therefore, breakpoints as well as clinical findings need to be precisely defined for differential diagnosis.

**Results:**

Chromosomes of a young female were analyzed due to primary amenorrhea, short stature, developmental delay and a characteristic facial appearance. Cytogenetic analysis using GTG banding identified a karyotype 46, XX, add(9pter). Surprisingly the application of high resolution molecular cytogenetic techniques characterized a partial trisomy 9p24.2-p22 and partial monosomy 9pter-p24.2. To the best of our knowledge only four similar case were reported by now.

**Conclusion:**

Attempts for genotype-phenotype correlations for partial trisomy 9p might have been hampered by the fact that more complex, cryptic aberrations were neither considered nor detected in comparable clinical cases.

## Background

Trisomy 9p is the fourth most frequent chromosome anomaly in life-born after trisomy 21, 18 and 13. A possible explanation might be that these chromosomes as well as 9p are relatively gene poor [1-3]. The first case of trisomy 9p was described in 1970 [1]. Since then, more than 150 patients with partial or complete trisomy 9p have been reported and this kind of chromosomal imbalance was characterized as a clinically recognizable syndrome. In most patients, the trisomic segment was transmitted from a parent carrying a reciprocal balanced translocation and only a small number arose from de novo duplications [3]. Rearrangements involving the distal region of the short arm of chromosome 9 (9p22 to 9p24) are well described and may involve deletions or duplications resulting in the partial monosomy [4] or the partial trisomy 9p syndrome [3]. Characteristic clinical features of partial trisomy 9p are mental retardation of various degree, short stature, craniofacial abnormalities, short fingers, simian crease, and single crease of the fifth finger. Additional symptoms like microcephaly, cleft lip and palate, malformed ears, and skeletal, nail, cardiac, and genital anomalies have also been observed [3]. Partial monosomy 9p or deletion 9p is also reported to be associated with a well defined phenotype characterized by mental retardation, flat occiput, trigonocephaly, inner epicanthal folds, mild hypertelorism, strabismus, flat nasal bridge, long philtrum, high arched palate, and flat feet [4].

In clinical cytogenetics, the precise identification of the chromosomal abnormality is a key factor when considering genotype-phenotype correlation. Advances in molecular cytogenetics have allowed more precise analysis of complex chromosomal rearrangements, especially with FISH techniques, spectral karyotyping, conventional comparative genomic hybridization (CGH) and FISH-banding [5]. The possibilities of array-CGH also refined the accuracy of characterization of complex chromosomal anomalies, such as unbalanced intrachromosomal rearrangements in general [3].

Here we report a new case of partial trisomy 9p involving cryptic monosomy of 9pter and discuss the possible impact of the detection of this case on the genotype-phenotype correlation in trisomy 9p.

## Case report

**Figure 1:**
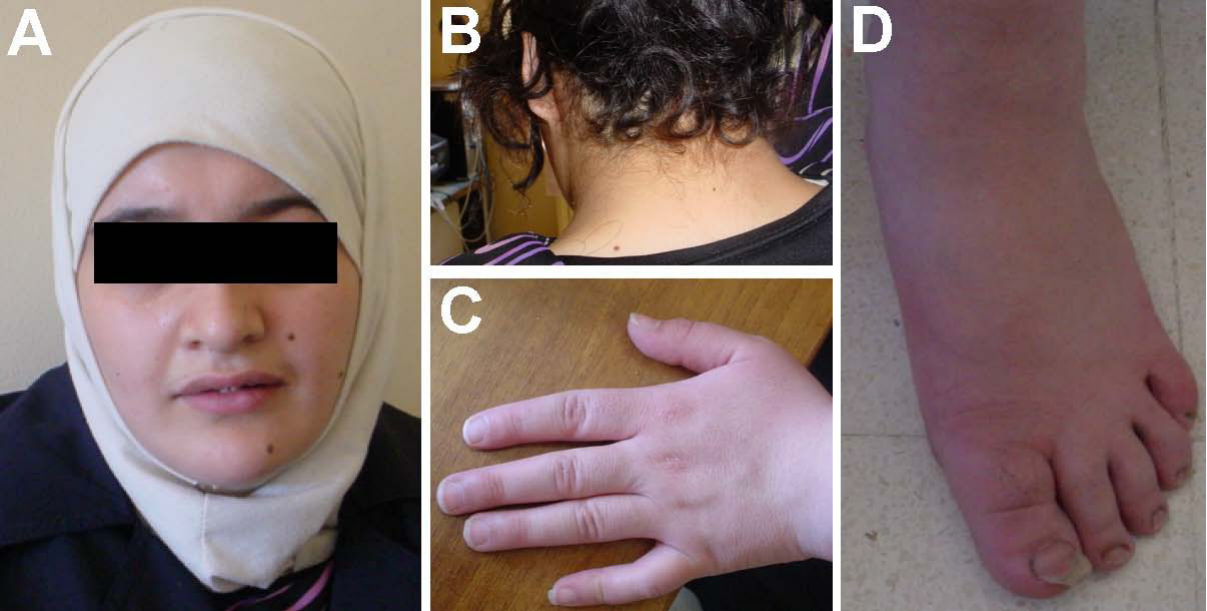
**Typical phenotypical feature of the present patient**: A) Facial appearance. B) Short neck and low set ears. C) Hand with short thumb and 5th finger (present bilateral). D) Malformed nails on toes and flat feet (present bilateral)

The patient, a 20-year-old female, was the first child of healthy non-consanguineous 26-year old mother and 33-year-old father. The girl was born at 37th weeks of gestation with a birth weight of 3,500 g; birth length and head circumference were not recorded. She was referred to cytogenetic analysis at age of 20 years due to a primary amenorrhea. Clinical examination revealed short stature (142 cm), IQ at the lower limit of normal, growth and developmental delay, a characteristic facial appearance (downturned mouth, bulbous nose, short philtrum, wide neck, low set ears, deep set eyes, thick lips), single crease of the fifth finger, short thumb and fifth finger (bilateral), flat feet and malformed toe-nails (see Fig. 1). The bone age was 16 years and the external genitalia were normal.

Ultrasound of pelvis revealed small uterus (5 × 2.5 × 2 cm) with large ovaries (46 to 47 mm) and polycystic ovaries. Hair distribution on the pubic is PHIII type, breast development is BIII type. Hormone levels: FSH 3.12 (normal up to 3.5-3.0 IU/l), LH 1.46 (normal up to 25 IU/l) and prolactin 45.3 (normal up to 29 ng/ml). The primary amenorrhea was treated by hormonal medication, leading to menstruation, which continued after drug was taken.

**Figure 2:**
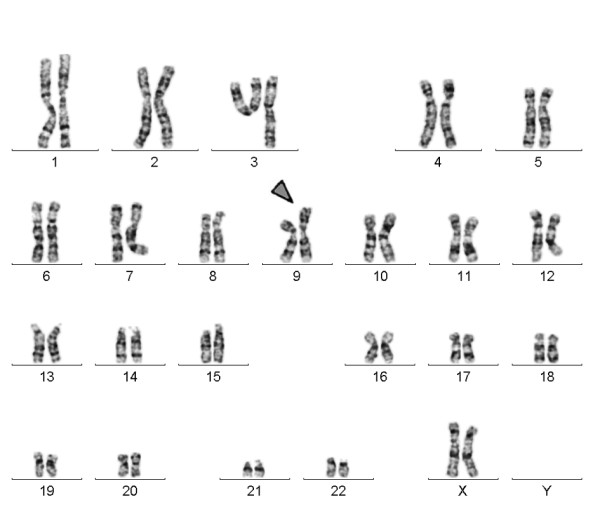
**GTG-banding revealed a 46, XX, add(9pter); a duplication of 9p material was suggested**. The derivative chromosome is marked by an arrowhead.

**Figure 3:**
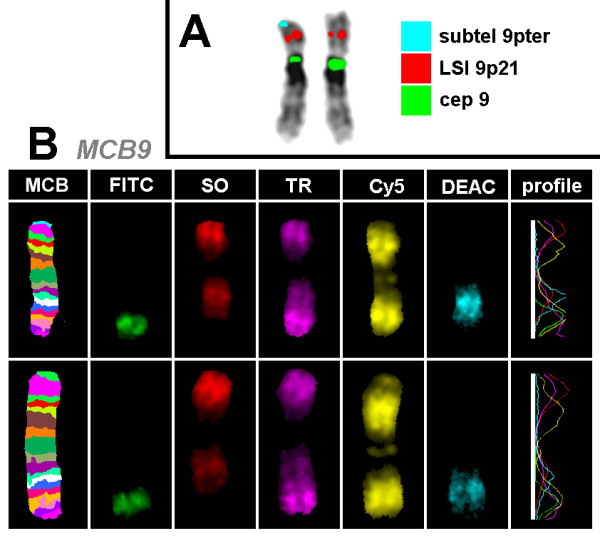
**Karyotype and chromosomal aberrations were confirmed using molecular cytogenetic approaches**. (A) A deletion on derivative chromosome 9pter was identified using the subtelomeric probe 9pter; application of the probe LSI 9p21 revealed that the breakpoint in the derivative chromosome 9 was distal from 9p21; the CEP 9 probe was used as a control. (B) The application of aMCB(9) characterized the additional material on derivative as chromosome 9 derived material and characterized the duplication of chromosome 9 in more detail.

Banding in conventional cytogenetics detected a karyotype of 46, XX, add(9pter) in all cells (Fig. 2). This finding was further studied by molecular cytogenetics; applying aMCB [6] and the subtelomeric probe specific for 9p (Fig. 3) the following result was obtained: 46, XX, der(9)(:p22->p24.2::p24.2- > pter) (20). The karyotypes of the mother and the father were 46, XX and 46, XY respectively.

## Discussion

Partial trisomy 9p was reported in numerous cases. It was seen as a result of different types of translocations, either de novo or inherited ones. The high frequency of the partial trisomy 9p may indicate a particular breakpoint sensitively of one or more regions of chromosome 9p [1,3,7].

Here we present the first case having both: partial trisomy 9p24.2-p22 and partial monosomy 9pter-p24.2. Symptoms typical for partial trisomy 9p were in the present patient short stature, short fingers and single crease of the fifth finger and nail anomalies. The finding of flat feet is more characteristic for partial monosomy of 9p. Typically observed in both conditions are mental retardation and craniofacial abnormalities. A similar case with partial trisomy 9p24.2-p21.3 and partial monosomy 9pter-p24.2 was recently reported [3]. There, as possibly specific features for this 'duplication/deletion 9p' condition, cleft palate and large eyes were discussed. However, both clinical features were absent in our patient. Besides, three other patients with partial trisomy 9p22.2-p13.3/partial monosomy 9pter-p22.2 [8], partial trisomy 9p22.3-p12/partial monosomy 9pter-p22.3 [8], and partial trisomy 9p22-p13/partial monosomy 9pter-p22 [9] were published. For all these cases the derivative chromosome 9 finishes with apparently with an open break, similar as in comparable cases a neo-telomere formation might be considered here, as well [10].

The phenotypic effects of different trisomic regions of chromosome 9p were reviewed [3] comparing the cytogenetic and clinical findings of ~150 previously published cases with partial or complete trisomy 9p. The majority of these cases were caused by an unbalanced translocation involving other chromosomes, as well. The impact of these additional segments on the phenotype was hard to be determined. Furthermore, the so-called characteristic trisomy 9p phenotype showed the best correlation with the 9p22 region [3]. Thus, the involvement of region 9p22 to9p24 in the present patient is in concordance with the general phenotype features of the partial trisomy 9p syndrome with mental retardation, developmental delay, short stature, characteristic facial appearance.

The critical region for viable partial deletions of the short arm of chromosome 9 seems to be relatively small. Up to present only comparatively minor terminal deletions of 9p, having relatively unspecific clinical signs, are reported [4,11]. Thus, overall, more cases characterized as having partial trisomy 9p syndrome might be found to be more complex than initially thought when analyzing them by FISH-banding or array-CGH approaches.

## Materials and methods

### Chromosome analysis

Chromosome analysis using GTG-banding was done according to standard procedures [12]. A total of 20 metaphases analyzed from stimulated peripheral blood culture were analyzed. Karyotypes were described according to the International System for Human Cytogenetic Nomenclature [13].

### Molecular cytogenetics

Fluorescence in situ hybridization (FISH) was done according to manufacturer's instructions using a locus specific probe LSI 9p21, a subtelomeric (ST) probe for the short arm of chromosome 9 and a chromosome enumeration probe (CEP) 9 (Abbott Molecular/Vysis, USA). An array-proven multicolor banding probe (aMCB) set for chromosome 9 was applied as described [6,14]. A total of 20 metaphase spreads were analyzed, each using a fluorescence microscope (AxioImager.Z1 mot, Zeiss) equipped with appropriate filter sets to discriminate between a maximum of five fluorochromes and the counterstain DAPI (4',6- diamino-2-phenylindole). Image capturing and processing were carried out using an ISIS imaging system (MetaSystems, Altlussheim, Germany) for the evaluation of aMCB.

## Competing interests

The authors declare that they have no competing interests.

## Authors' contributions

AW and FM performed the cytogenetic studies in the present case and collected the data relative to this case report. WA supervised the cytogenetic analysis as Director of the HGD. AW, FM, TL did the molecular cytogenetic analysis and interpretation. TL drafted the paper, and all authors contributed to the finalizing of, and read and approved the final manuscript.

## Consent

Written informed consent was obtained from the patient for publication of this case report and accompanying images. A copy of the written consent is available for review by the Editor-in-Chief of this journal.

## References

[B1] Rethoré MO Larget-Piet L Abonyi D Boeswillwald M Berger R Carpentier S Cruveiller J Dutrillau B Lafourcade J Penneau M Lejeune J [4 cases of trisomy for the short arm of chromosome 9. Individualization of a new morbid entity] Ann Genet 1970 13 217 232 5313386

[B2] Krepischi-Santos AC Vianna-Morgante AM Disclosing the mechanisms of origin of de novo short-arm duplications of chromosome 9 Am J Med Genet 2003 117A 41 46 10.1002/ajmg.a.10634 12548739

[B3] Hulick PJ Noonan KM Kulkarni S Donovan DJ Listewnik M Ihm C Stoler JM Weremowicz S Cytogenetic and array-CGH characterization of a complex de novo rearrangement involving duplication and deletion of 9p and clinical findings in a 4-month-old female Cytogenet Genome Res 2009 126 305 312 10.1159/000251966 20068300 PMC3711006

[B4] Huret JL Leonard C Forestier B Rethore MO Lejeune J Eleven new cases of del(9p) and features from 80 cases J Med Genet 1988 25 741 749 10.1136/jmg.25.11.741 3070043 PMC1051577

[B5] Liehr T Starke H Weise A Lehrer H Claussen U Multicolor FISH probe sets and their applications Histol Histopathol 2004 19 229 237 14702191 10.14670/HH-19.229

[B6] Weise A Mrasek K Fickelscher I Claussen U Cheung SW Cai WW Liehr T Kosyakova N Molecular definition of high-resolution multicolor banding probes: first within the human DNA sequence anchored FISH banding probe set J Histochem Cytochem 2008 56 487 493 10.1369/jhc.2008.950550 18256020 PMC2324187

[B7] Krepischi-Santos AC Vianna-Morgante AM Disclosing the mechanisms of origin of de novo short-arm duplications of chromosome 9 Am J Med Genet 2003 117A 41 46 10.1002/ajmg.a.10634 12548739

[B8] Swinkels ME Simons A Smeets DF Vissers LE Veltman JA Pfundt R de Vries BB Faas BH Schrander-Stumpel CT McCann E Sweeney E May P Draaisma JM Knoers NV van Kessel AG van Ravenswaaij-Arts CM Clinical and cytogenetic characterization of 13 Dutch patients with deletion 9p syndrome: delineation of the critical region for a consensus phenotype Am J Med Genet A 2008 146A 1430 1438 10.1002/ajmg.a.32310 18452192

[B9] Teebi AS Gibson L McGrath J Meyn MS Breg WR Yang-Feng TL Molecular and cytogenetic characterization of 9p-abnormalities Am J Med Genet 1993 46 288 292 10.1002/ajmg.1320460310 8488873

[B10] Kulikowski LD Christ LA Nogueira SI Brunoni D Schwartz S Melaragno MI Breakpoint mapping in a case of mosaicism with partial monosomy 9p23 --> pter and partial trisomy 1q41 --> qter suggests neo-telomere formation in stabilizing the deleted chromosome Am J Med Genet A 2006 140 82 87 16333825 10.1002/ajmg.a.31045

[B11] Techakittiroj C Kim KC Andersson H Li MM 9p subtelomere deletion: pathogenic mutation or normal variant? Beijing Da Xue Xue Bao 2006 38 92 93 16415976

[B12] Claussen U Michel S Mühlig P Westermann M Grummt UW Kromeyer-Hauschild K Liehr T Demystifying chromosome preparation and the implications for the concept of chromosome condensation during mitosis Cytogenet Genome Res 2002 98 136 146 10.1159/000069817 12697995

[B13] Shaffer L Slovak M Cambell L (eds) ISCN (2009): An International System for Human Cytogenetic Nomenclature Karger Basel 2009

[B14] Liehr T Heller A Starke H Rubtsov N Trifonov V Mrasek K Weise A Kuechler A Claussen U Microdissection based high resolution multicolor banding for all 24 human chromosomes Int J Mol Med 2002 9 335 339 11891523

